# Supplementary material for: Functional Characterization of Largemouth Bass (Micropterus salmoides) Soluble FcγR Homolog in Response to Bacterial Infection
Source: Int J Mol Sci. 2022 Nov 9;23(22):13788. doi: 10.3390/ijms232213788 (PMC9699129; doi:10.3390/ijms232213788)
Supplement: Supplementary file 1 [file ijms-23-13788-s001.zip › ijms-1930648-supplementary.pdf]

Supplementary materials

Table S1. Primers used in this study.

Primer name	Sequence (5′-3′)	Used	Source
MsFcγRIα-O F	ATGGACACGGTCATTTCCCTCCT	Clone of FcγRIα ORF	OK258092.1
MsFcγRIα-O R	CCATAGTAAAGTGTCTGGAGAGTCAT		
MsFcγRIα-32a F	**CGC**GGATCCGATGCTCCCTCTGTGACCTCATT	Clone of FcγRIα-PET32a	OK258092.1
MsFcγRIα-32a R	**GGG**AAGCTT**G**CCATAGTAAAGTGTCTGGAGAGTC		
MsFcγRIα-EGFP F	**GGG**AAGCTTATGGACACGGTCATTTCCCTCCT	Clone of FcγRIα-PEGFP-N1	OK258092.1
MsFcγRIα-EGFP R	**CGC**GGATCC**GG**CCATAGTAAAGTGTCTGGAGAGTC		
MsFcγRIα-3.1 F	**CCC**AAGCTTGCCACCATGGACACGGTCATTTCCCTCCT	Clone of FcγRIα-PcDNA3.1(+)	OK258092.1
MsFcγRIα-3.1 R	**CGC**GGATCCTTACCATAGTAAAGTGTCTGGAG		
qFcγRIα F	GGTGCCCGAGTTAGACA	qRT-PCR	[60]
qFcγRIα R	GGAGGACCTGGATTAGATT		
qLyn F	TTATGTTGCCCAAGTTGACACC	qRT-PCR	[60]
qLyn R	TGTTTGATCATGCTGCCGAT		
qSyk F	ATGGAGAACTGGGCTCT	qRT-PCR	[60]
qSyk R	CTATTTGTATGCAGGTGTAATGTGA		
qβ-actin F	CCACCACAGCCGAGAGGGAA	qRT-PCR	[60]
qβ-actin R	TCATGGTGGATGGGGCCAGG		
qCFL F	CACGCAGACGCTGACCT	qRT-PCR	[60]
qCFL R	:CCTGAGCCTTACGCACC		
qArp2/3 F	TGAAGAACCCGCCAATCAACACA	qRT-PCR	[60]
qArp2/3 R	CCAGAGCCTTTTCATGCCACT		
qATG3 F	AAATGGTAGAAACTAAAGCCAA	qRT-PCR	this study
qATG3 R	TATCCAAACAGCCAGAGTCGAG		
qMARCKS F	GGAGCGACAGCAGAAGAAC	qRT-PCR	[60]
qMARCKS R	TGCCACCTTCACCACCTC		
qC1R F	TCCCAGACAATGAGACC	qRT-PCR	this study
qC1R R	GGCAGACGGACAAACCT		
qC3 F	GGTCAGTTGGTGGATAAA	qRT-PCR	this study
qC3 R	TGATACGGAATGATGGAA		
qC5 F	GTCAGCTGGATAAGAGCGTAAG	qRT-PCR	[61]

qC5 R	GTCGGTCTGGATAAACAGGAAG		
qC7 F	CGTCGCACTTTCCACAA	qRT-PCR	this
qC7 R	CTTCGGACAAGGTCAGATA		study
qmIgM F	AAGGCCGGTTTACCATC	qRT-PCR	this
qmIgM R	AAGATTATTATACCTCGAGCAC		study

Note： Bold letters represent protective base, underline letters represent restriction sites and red letters represent Kozak sequence.

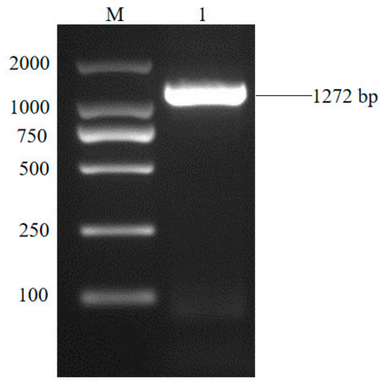

Figure S1. Agarose gel electrophoresis of PCR product of *MsFcγRIα* gene. Lane M: DL2000 marker; Lane 1: open reading frame of *MsFcγRIα* gene.

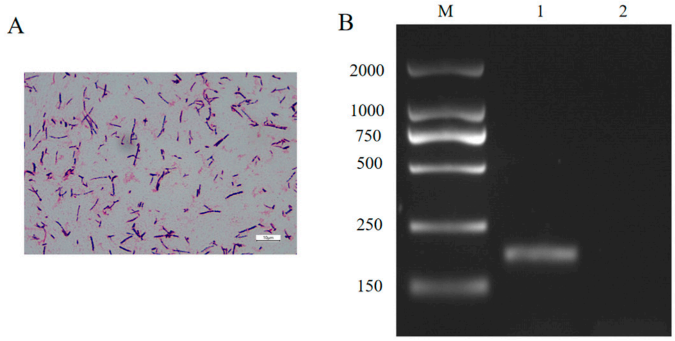

Figure S2. Identification of *N. seriolae*. A: Result of Gram's stain; B: PCR of *N. seriolae*; Line M: DL2000 marker; Lane 1: *N. seriolae*; Line 2: Negative control.

References

[60]Wu, J.; Nie, Y.; Ma, Y.; Hao, L.; Liu, Z.; Li, Y. Analysis of phagocytosis by mIgM+ lymphocytes depending on monoclonal antibodies against IgM of largemouth bass (*Micropterus salmoides*). *Fish Shellfish Immunol.* **2022**, *123*, 399–408. https://doi.org/10.1016/j.fsi.2022.03.014.

[61] Yang, C.,, Dong, J., Sun, C., Li., W., Tian, Y., Liu, Z., Gao. F., Ye, X. Exposure to heat stress causes downregulation of immune response genes and weakens the disease resistance of Micropterus salmoides. *Comparative biochemistry and physiology. Part D, Genomics & proteomics* **2022**, 43, 101011. https://doi:10.1016/j.cbd.2022.101011.
